# Spatial accessibility to health facilities in Sub-Saharan Africa: comparing existing models with survey-based perceived accessibility

**DOI:** 10.1186/s12942-022-00318-z

**Published:** 2022-11-12

**Authors:** Jérémie Bihin, Florence De Longueville, Catherine Linard

**Affiliations:** 1 Department of Geography, University of Namur, 5000 Namur, Belgium; 2 ILEE, University of Namur, 5000 Namur, Belgium; 3 NARILIS, University of Namur, 5000 Namur, Belgium

**Keywords:** Access to health, Spatial modelling, Perceived accessibility, Sub-Saharan Africa

## Abstract

**Background:**

Mapping geographical accessibility to health services is essential to improve access to public health in sub-Saharan Africa. Different methods exist to estimate geographical accessibility, but little is known about the ability of these methods to represent the experienced accessibility of the population, and about the added-value of sophisticated and data-demanding methods over simpler ones. Here we compare the most commonly used methods to survey-based perceived accessibility in different geographical settings.

**Methods:**

Modelled accessibility maps are computed for 12 selected sub-Saharan African countries using four methods: Euclidean distance, cost-distance considering walking and motorized speed, and Kernel density. All methods are based on open and large-scale datasets to allow replication. Correlation coefficients are computed between the four modelled accessibility indexes and the perceived accessibility index extracted from Demographic and Health Surveys (DHS), and compared across different socio-geographical contexts (rural and urban, population with or without access to motorized transports, per country).

**Results:**

Our analysis suggests that, at medium spatial resolution and using globally-consistent input datasets, the use of sophisticated and data-demanding methods is difficult to justify as their added value over a simple Euclidian distance method is not clear. We also highlight that all modelled accessibilities are better correlated with perceived accessibility in rural than urban contexts and for population who do not have access to motorized transportation.

**Conclusions:**

This paper should guide researchers in the public health domain for knowing strengths and limits of different methods to evaluate disparities in health services accessibility. We suggest that using cost-distance accessibility maps over Euclidean distance is not always relevant, especially when based on low resolution and/or non-exhaustive geographical datasets, which is often the case in low- and middle-income countries.

## Introduction

Mapping spatial accessibility to health facilities is an essential methodological challenge in public health research and in global welfare monitoring [9, 10]. The SARS-CoV-2 pandemic that started in the end of 2019 highlighted the necessity of producing accessibility maps to health facilities in order to identify inhabited regions that are spatially isolated from health care systems [8]. Because geographic accessibility to health facilities has a direct impact on the quantity of preventive or curative health services provided [15, 28, 29], it also affects health outcomes in case of injury or disease [10, 14, 17]. However, accessibility maps are estimations and may fail at representing the experienced accessibility of the population. Errors from accessibility maps may be more important in low- and middle-income regions when input data on health facility and population location are lacking. Yet, those low- and middle-income regions such as sub-Saharan Africa (SSA) also have the biggest disparities in accessibility to health services [26].

Penchansky et al. [20] defined accessibility to health services as “*the relationship between the location of supply and the location of clients, taking account of clients transportation resource and travel time, distance and cost*.” This definition includes spatial factors such as proximity between patients and health practitioners, road availability, and quality or traffic, but also individual factors such as access to motorized transportation, financial ability to pay for public transportation or health condition adequacy for traveling [4]. Accessibility mapping methods generally consist in GIS-based quantitative estimations of spatial factors, but it is harder to take individual factors into account.

Accessibility mapping methods can be of various complexity levels, from a simple Euclidean distance between the population location and the closest health facility (= straight-line distance) to complex travel-time estimation to the closest health facility, based on a cost-distance algorithm. The latter method takes various ancillary data into account, such as relief, road infrastructures or land-cover [12]. Weiss, D.J. et al. [26]. Other methods like Kernel density or the enhanced two-step floating catchment area consider multiple health facilities, generating a higher accessibility index if multiple health facilities are close by, rather than only considering the closest one [18]. More complex methods are developed to better represent the experience of the population. For example, the cost-distance method is able to take geographical obstacles and road infrastructure into account, while the Euclidean distance does not. However, it is difficult to quantify the extent to which a complex method improves estimates over a simpler method, or even if it improves them at all. These different measures of accessibility were shown to be highly correlated in a developed country such as the US [5], making the benefit of the cost-distance method quite small compared with simpler methods for non-emergency travel to hospitals. Yet, the difference between these methods could be more important in countries where the road network is more heterogeneous and geographical obstacles are more frequent. It is therefore essential to quantify how methods differ in predicting the experienced accessibility to health facilities in different contexts.

The most straightforward way to compare accessibility mapping methods is to confront modelled accessibility measures with experienced accessibility. Experienced accessibility cannot be measured directly, but it can be estimated using proxy data such as the use of a specific health service in a population [3, 29]. However, data on health service use can be difficult to collect, especially in low-income countries. Survey-based perceived accessibility may also be used as a proxy to experienced accessibility, as it is an important factor in the decision of whether to seek health care [2]. Perceived accessibility may therefore be used to validate accessibility models [7]. Survey questions can, amongst other information, collect data on patients’ satisfaction with their access to health services [4] or on barriers that prevent the population from accessing health services [15]. These data can be compared with modelled accessibility results to see which model correlates better with the population perception, as Baier et al. [4] did in their research in Germany. As far as we know, such research has not yet been conducted in low- and middle-income countries.

The objective of this paper is to confront four different accessibility mapping methods in 12 countries of SSA, by comparing their correlation values with survey-based perceived accessibility. The selected methods are: (1) Euclidean distance to the closest health facility, (2) Cost-distance to the closest health facility, considering motorized speed, (3) Cost-distance to the closest health facility, considering walking speed, and (4) Kernel density of health facilities. As we expect the geographical context to impact the performance of the methods, we stratified analyses by country, urban/rural context, and access to motorized transportation. We focus our analyses on mapping methods that are easily applicable globally, with easy access to data, not on local methods that require field expertise and intense local data collection.

## Material and methods

Twelve SSA countries were selected for this study, based on data availability (Table 1). These countries are diverse in terms of size, population density, and landscape.

**Table 1:** General statistics of DHS datasets used in this analysis

Country name	Year(s) of the survey data collection	N. respondents	N. clusters	N. health facilities	% of respondents seeing distance as an obstacle when medical treatment needed	% of respondents living in a rural environment	% of respondents motorized
Angola	2015–2016	14379	624	1450	51.29	37.86	27.87
Benin	2017–2018	15928	537	823	30.93	55.77	62.82
Burundi	2016–2017	17269	552	666	33.50	78.74	5.11
Ethiopia	2016	15683	622	5175	44.70	65.89	3.43
Guinea	2018	10874	400	1519	47.53	62.83	32.48
Malawi	2015–2016	24562	849	642	51.09	78.64	6.44
Mali	2018	10519	328	1449	30.90	66.56	58.40
Nigeria	2018	41821	1358	19695	27.39	59.39	40.85
Rwanda	2014–2015	13497	492	573	20.68	74.61	4.67
Tanzania	2015–2016	13266	603	6452	39.86	68.75	14.63
Uganda	2016	18506	685	3678	39.98	76.34	14.42
Zimbabwe	2015	9955	399	1201	29.90	54.59	17.72
Total	–	206 259	7449	43323	36.75	65.45	24.61

### GIS data

Geolocated health facilities were extracted from a database assembled by Maina et al. [19], composed of 98,745 geocoded public health facilities in SSA. They used a data compilation, geocoding, cleaning and validation process based on multiple data sources, with a focus on official sources from Ministries of Health, and completed with non-governmental data when necessary.

Friction maps were necessary to generate travel-time estimations with the cost-distance method. Friction maps represent the ease to travel in a given landscape. More specifically, they are raster grids where every cell contains a value representing the time necessary to cross the pixel. These time values are estimated using geographical variables such as land cover, road network or relief. Friction maps are provided by the “malariaAtlas” package of the R software, which is developed by the Malaria Atlas Project team [21]. From this package, we extracted two 2019 friction maps: one considering that the population has access to motorized transportation, and the other considering walking speed only [26, 27]. Both have a spatial resolution of 30 arc seconds (~ 1 × 1 km at the equator). Administrative unit limits were also extracted from the malariaAtlas R package.

For general accessibility measures, we used gridded population datasets produced by WorldPop (www.worldpop.org), where every cell contains an estimation of population count. These maps are generated by disaggregating population count data from administrative units, provided by official national censuses, into 100 × 100 m raster cells. The disaggregation is done using a semi-automated machine learning method based on a random-forest model, and spatial ancillary data such as road network, built areas, land-cover etc. [23]. We used the continental-wide population map provided at a spatial resolution of 0.00833 decimal degrees (~ 1 km at the equator) in order to match the friction maps and reduce computation needs.

### Survey data

The Demographic and Health Surveys (DHS; http://www.dhsprogram.org) are standardized surveys, conducted in 90 countries, that collect data on health, wealth, education and household characteristics. DHS respondents living in the same neighborhood are aggregated by clusters. For the sake of confidentiality, GPS coordinates of the cluster centroid are subject to a displacement of a random value between 0 and 2 km for urban clusters and between 0 and 5 km for rural clusters, in a random direction. One percent of the rural clusters receive an additional displacement of a distance between 0 and 10 km. Those displacements are however constrained within the administrative unit level 2 of the country. For the majority of DHS surveys, GPS coordinates of the dislocated cluster centroid are provided. Here we selected 12 SSA countries where surveys have been conducted after 2015 and for which GPS coordinates are available (Table 1).

For the present study, we are particularly interested in the perceived accessibility to health facilities, collected in the DHS individual surveys for women. The question v467 from that survey asks the following: “When you are sick and that you need medical advice or treatment, are the following elements an obstacle to you, yes or no” and the fourth option, coded as v467d, is “Distance to the health facility”. To estimate the perceived accessibility of the population, we used the proportion of women per cluster who have answered “Yes” at the question v467d. This variable is hereafter called PA (Perceived Access). We identified the respondents having access to personal motorized transportation with the questions hv211 and hv212 from DHS, asking respectively if any member of the household owns a motorcycle or a car. Table 1 presents a summary of the data extracted from the DHS surveys.

### Modelled accessibility

The goal of this paper is to compare four commonly used spatial models of accessibility with survey-derived perceived accessibility. Each modelling method produces a raster grid with an accessibility value for every cell.

- The Euclidean Distance (ED) method computes the on-the-fly distance value to the closest health facility in meters, for each cell of a raster grid, using the “distanceFromPoints” tool from the “raster” package in the R software [11].
- The Cost-Distance Motorized (CD-M) method uses a friction map and a cost-distance algorithm in order to generate a raster grid, with an estimated travel-time value in minutes for each cell. This method uses the friction map considering access to motorized transportation. We used the function “accCost” from the “gdistance” package in R [25], following the method and code from Weiss et al. [27].
- The Cost-Distance Walking (CD-W) method is identical to CD-M, except that it is based on the friction map considering walking speed only.
- The Kernel Density (KD) method computes a point-density continuous surface based on the geolocated health facilities. It generates a dimensionless accessibility value for each cell, using a common Gaussian impedance function, with a distance threshold fixed at 15 km beyond which the accessibility value will be zero. In the absence of robust analyses on the best distance threshold to use, 15 km seemed reasonable here because it is a rather large distance to walk to obtain health care while avoiding a large proportion of the population being assigned a zero-access value. The accessibility values were then summed up for all health facilities, making the KD method the only one considering multiple health facilities in the vicinity to evaluate accessibility. This method generates values varying between 0 and ~ 1.5. Note that the KD method produces values that are higher when accessibility is also higher, contrarily to the other methods.

The four accessibility maps were cropped and resampled in order to match the same extent and cell size as the population raster grid of their respective country. For each geolocated DHS cluster, we extracted PA and the four modelled accessibility values (MAs), i.e. ED, CD-M, CD-W and KD.

### Statistical analyses

First, we measured Spearman correlation coefficients between the different modelling methods, in order to evaluate the degree of similarity of their estimates. Given that Kernel density does not have a linear relationship with the three other methods, the Spearman index, which measures the correlation based on variable ranks, is more appropriate.

We then measured Spearman correlation coefficients between PA and MAs, first taking all data together, and then stratifying them into subgroups: (i) by socio-geographic context, i.e. by isolating respondents living in rural or urban areas, and by isolating respondents having access to motorized transportation or not, (ii) by country. Our exploratory analysis and previous studies suggest that non-linear functions such as logistic function or hyperbolic decline better explain the relationship between distance or travel time and perceived accessibility [13, 16]. Scatterplots representing the statistical relationship between PA and MAs were computed and a local weighted smoothing method was used to help visualizing the shape of the relationships.

Because the displacement of DHS clusters may influence MA values, we estimated the range of variability induced by such displacements by artificially moving cluster points 30 times within buffer zones of the same size, as the ones used by DHS (2 km radius for urban clusters, 5 km radius for rural clusters). For each virtual displacement, new MA values were extracted and new Spearman correlation coefficients were calculated.

Finally, the overall accessibility of the population to health facilities was calculated and compared for the 12 countries by overlaying MA maps with gridded population maps. We extracted the proportion of the population living in different distance classes for the Euclidean distance method or travel-time classes for cost-distance methods. These accessibility measures were not calculated with the Kernel density method because it generates dimensionless values that are not appropriate for this type of analysis.

## Results

Table 2 shows Spearman correlation coefficients between MA values, extracted at each DHS cluster location. Euclidean distance and both cost-distance methods are highly positively correlated (r_s_ > 0.84, p-values < 0.001). The Kernel Density method is negatively correlated to the three other MAs, with correlation indexes of − 0.553, − 0.607 and − 0.604 (p-value =  < 0.001). The Kernel density also present negative correlation results, which was expected since it is the only method producing values that are positively correlated to accessibility. Table 3 presents the correlation values between PA and MAs, first all data taken together, and then by rural/urban and motorized/non-motorized classes. Spearman correlation coefficients range from 0.085 to 0.457, with Euclidean distance always having the highest correlation coefficient compared to other methods, followed by cost-distance walking, cost-distance motorized and Kernel density. Correlation indexes are higher for rural respondents than urban ones, and for respondents without access to motorized transport compared to those who have access, whatever the method. This suggests that those models relate better to perceived accessibility in rural contexts with low access to motorized transportation.

**Table 2:** Spearman correlation coefficients (in absolute values) between the four methods, all clusters from all 12 countries aggregated

	Euclidean distance	Cost-distance motorized	Cost-distance walking	Kernel density
Euclidean distance	1	0.844	0.966	0.553
Cost-distance motorized		1	0.895	0.607
Cost-distance walking			1	0.604
Kernel density				1

**Table 3:** Spearman correlation coefficients (r_S_) between perceived accessibility (PA) and modelled accessibility (MA) in absolute values, first for all data, and then stratified by geographic context (urban or rural) and access to motorized transportation

All data aggregated			
		rS	
Euclidean distance		0.457	
Cost-distance motorized		0.424	
Cost-distance walking		0.444	
Kernel density		0.308	

Stratified by geographic context and transportation mean			
		rS	
		Urban	Rural
Euclidean distance	Motorized	0.191	0.260
Cost-distance motorized		0.172	0.204
Cost-distance walking		0.177	0.248
Kernel density		0.085	0.181
Euclidean distance	Non-motorized	0.239	0.369
Cost-distance motorized		0.216	0.309
Cost-distance walking		0.220	0.359
Kernel density		0.117	0.260

**Fig. 1:**
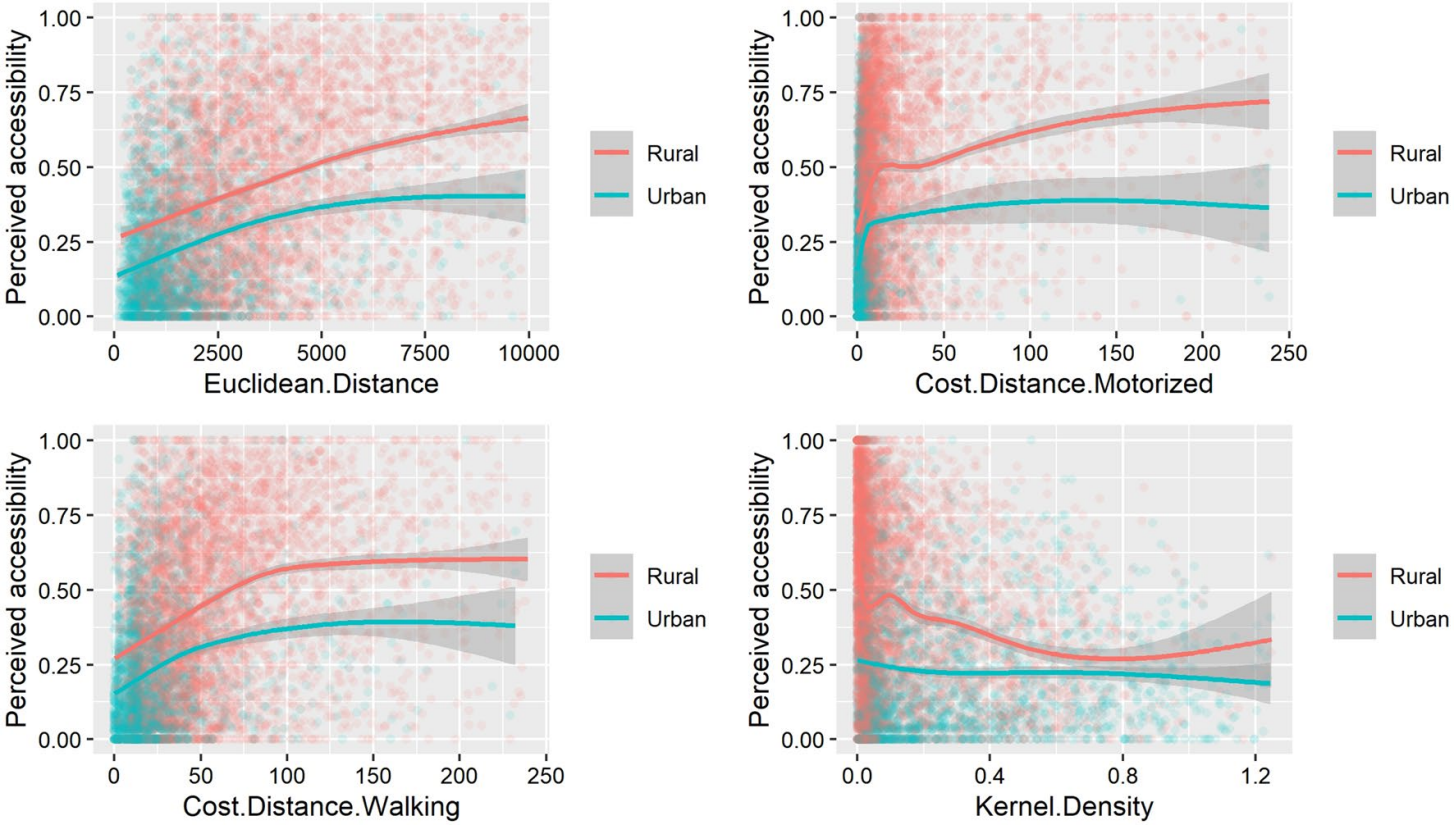
Relationship between modelled and perceived accessibility for rural and urban clusters. Euclidean distance **A** is expressed in meters, cost-distance methods **B**, **C** in minutes and Kernel density **D** in an adimensional unit. Curves are generated with a GAM function, with the shaded area showing confidence intervals

Figure 1 shows the relationship between MAs and survey-based PA stratified by urban and rural clusters. We can see that, for a given MA value (distance, travel time or Kernel index), the perception of respondents regarding their accessibility is worse in a rural context than in an urban one. As expected, Euclidean distance (Fig. 1A) and cost-distance methods (Fig. 1B, C) show a positive relationship between MA and PA. For the cost-distance motorized method (Fig. 1B), the slope is very abrupt early on, indicating that, for a given estimated travel-time increase, the proportion of respondents seeing distance as an obstacle increases rapidly. Globally, the relationship between Kernel density (Fig. 1D) and perceived accessibility is negative, which is expected since this model produces an accessibility value that is positively proportionate to accessibility, contrarily to the three other methods. We also observe for all methods that the relationship between PA and MA is not linear and that there is an important variability in PA results for a given accessibility value, suggesting that MA only explains a small share of the variability of PA.

**Fig. 2:**
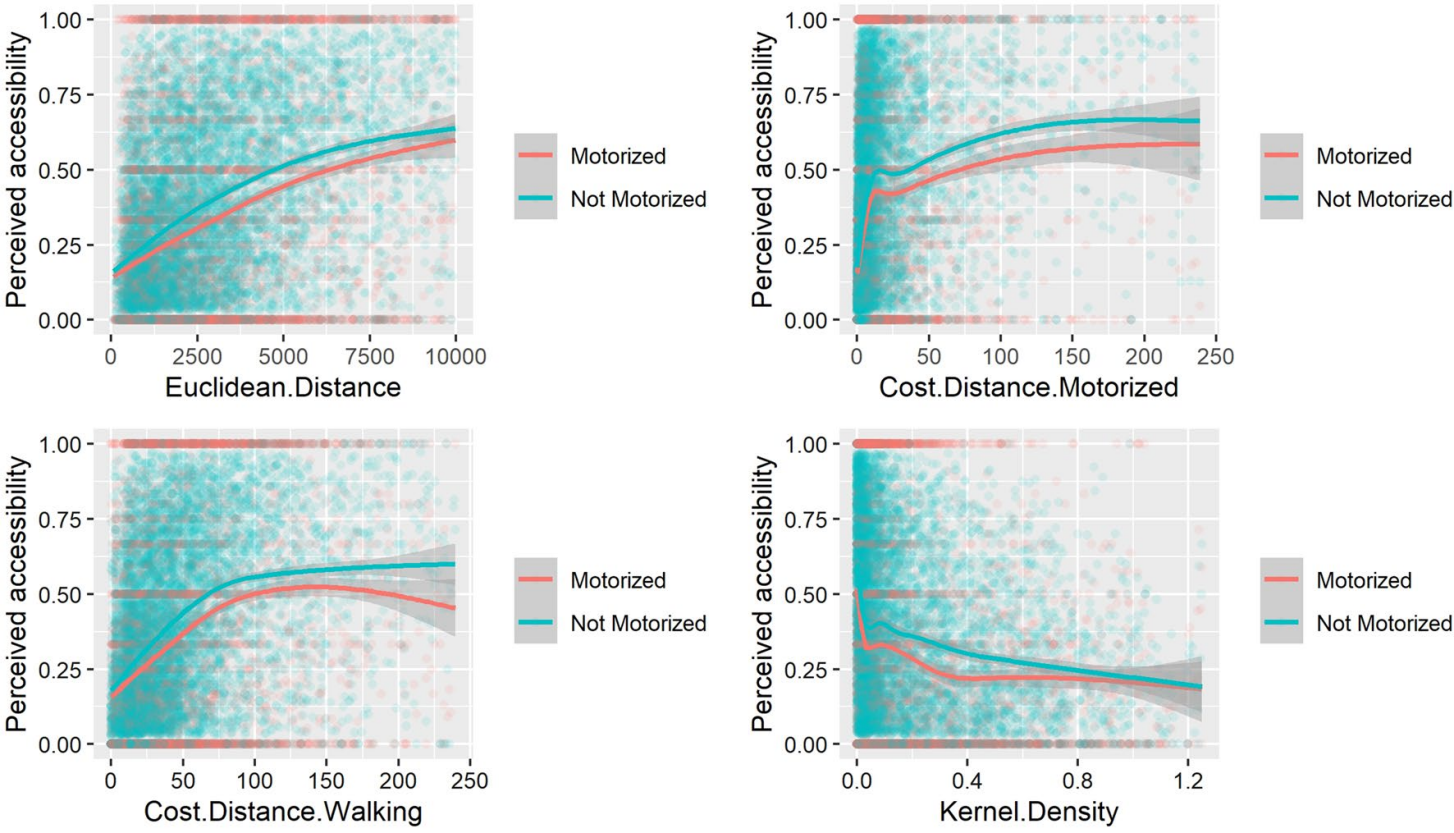
Relationship between modelled and perceived accessibility for respondents with or without access to motorized transportation. Euclidean distance **A** is expressed in meters, cost-distance methods **B**, **C** in minutes and Kernel density **D** in an adimensional unit. Curves are generated with a GAM function, with the shaded area showing confidence intervals

**Fig. 3:**
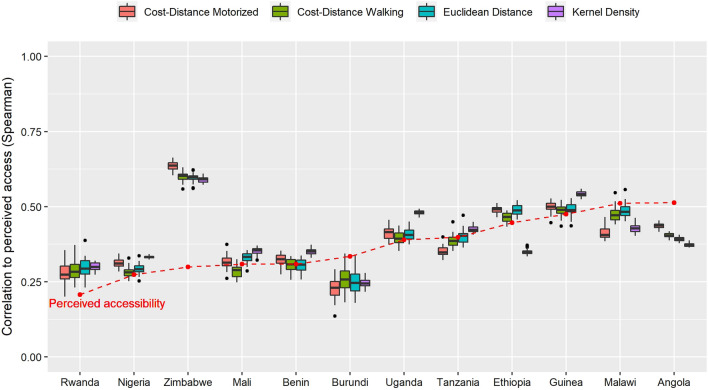
Distribution of Spearman correlation coefficients for 30 replications of randomly-displaced cluster points for each method and each country. Countries are ordered by increasing mean perceived accessibility (% of respondents identifying distance as an obstacle, represented by the red dotted line)

Figure 2 presents the relationships between MA and PA, separating respondents with or without access to motorized transportation. We can see that, for a given accessibility value, respondents without access to motorized transportation more often see distance as an obstacle when in need of health care. The relationships have a similar shape as in Figs. 1, 3 shows, for each country and each method, the variability of correlation coefficients between PA and MA for 30 replications of randomly-displaced geolocated cluster points. The majority (79.8%) of the correlation indexes lies between 0.25 and 0.50. Zimbabwe shows significantly higher correlation coefficients than the other countries, with a mean correlation of 0.61. We can see here that the Kernel density method has the best correlation values (p.value < 0.005) for six countries out of twelve, which brings nuance to the results of Table 3, where Kernel density seemed to have the worst correlation level with perceived accessibility.

The variability of correlation coefficients due to the point displacement process used here (boxplot sizes) is generally low compared to inter-country or inter-method variabilities. It suggests that the impact of the DHS point displacement process on correlation values is limited and do not invalidate our results. Nonetheless, 94.7% of the point displacements resulted in a lowering of correlation indexes. The Kernel density method is less sensitive to point displacements, with a mean absolute correlation difference (0.013) lower than for the three other methods (0.032–0.042). Similarly, small countries are more sensitive to point displacements than bigger countries, with the difference in Spearman correlation coefficients (r_S_) caused by the point displacement process positively correlated to the size of countries (r_S_^ED^ = − 0.804, r_S_^CD−M^ = − 0.692, r_S_^CD−W^ = -0.832 and r_S_^KD^ = − 0.447, p-values < 0.005).

**Fig. 4:**
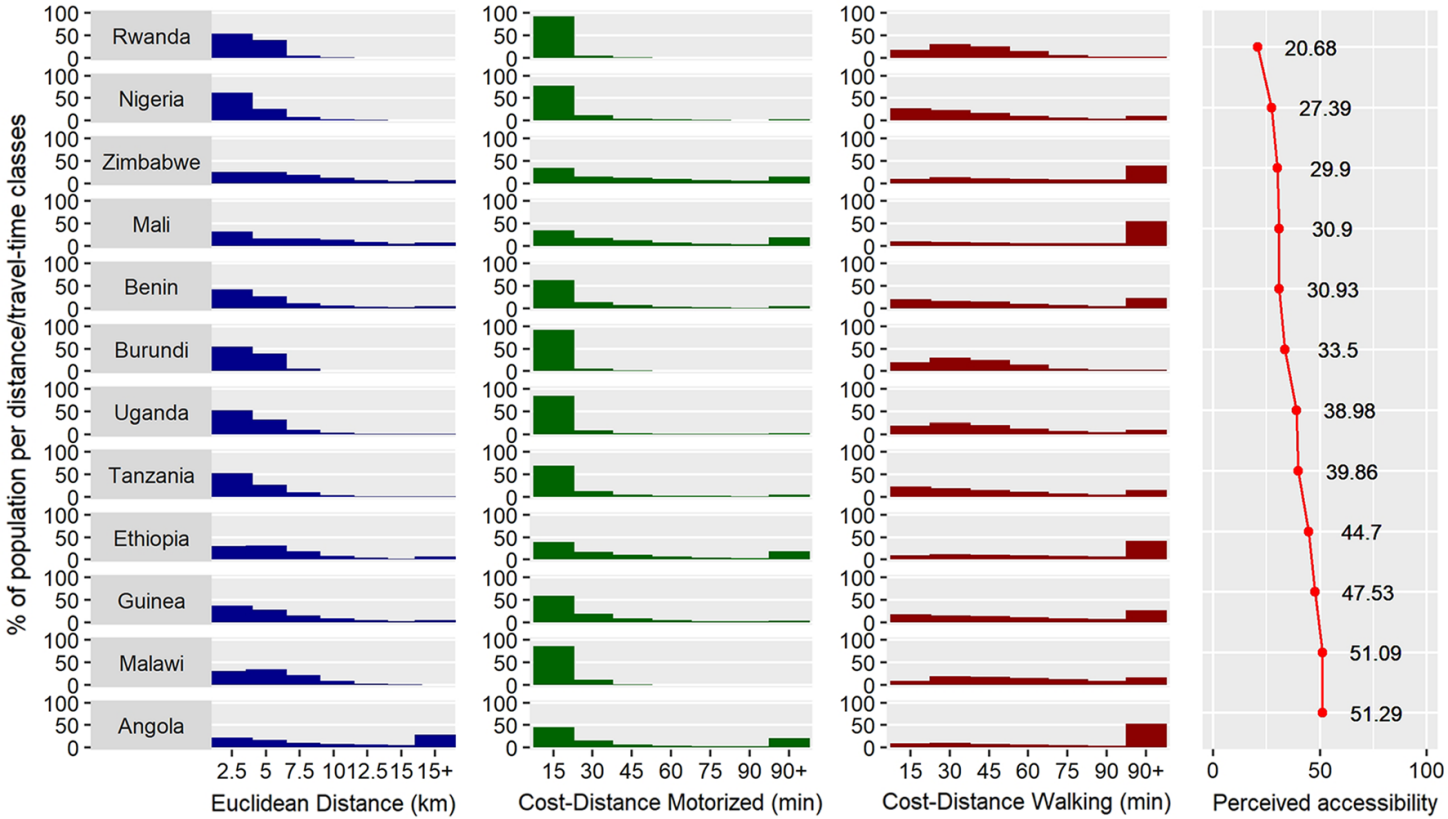
Distribution of population per accessibility class, for three different methods (ED, CD-M and CD-W). The column "Perceived accessibility" indicate the percentage of respondents per country indicating that distance is an obstacle when they need medical care

Figure 4 represents, for each country, the proportion of population that lives in different accessibility classes considering Euclidean distance, cost-distance motorized or cost-distance walking methods. On average, the worst PA is observed in Angola with 51.29% of the respondents considering that distance is an obstacle. In comparison, the average PA is 20.68% in Rwanda, making it the country with the best perceived accessibility results. These observations are confirmed by the accessibility of the population estimated by the ED method shown in Fig. 4: Angola has only 33.34% of its population living at less than five kilometers from the closest health center, and 28.09% at more than 15 km, where Rwanda has 81.04% of its population living at less than five kilometers, and 0.002% of its population at more than 15 km.

Figure 4 also shows that the choice of accessibility modelling method will strongly influence the conclusions that can be drawn about accessibility for a given population in a given country. For example, for Burundi, if we look at the first accessibility class for each method, results are strongly varying: considering Euclidean distance, 15.84% of the population lives at less than 1 km of the closest health facility. Considering cost-distance with motorized speed, 85.05% of the population lives at less than 10 min of the closest health facility, but if we consider cost-distance with walking speed, it decreases to only 5.73%. This brings out how the choice of an accessibility computation method and the choice of arbitrary accessibility class limits can strongly modify the conclusions drawn, when assessing accessibility disparities in a region.

## Discussion

The goal of this paper was to evaluate how different accessibility modelling methods relate to the perception of the population of their own accessibility to health facilities, and to compare those methods together. Underlying methodological goals were, on one hand, to compute those analyses using data and methods that are commonly used in the literature and easy to access and reproduce and, on the other hand, to evaluate the impact of DHS point displacement on our results.

Results from the correlation analyses, all countries taken together, indicate that accessibility models better explain the perception of the population in rural contexts than in urban ones. This result was expected since we suppose that mobility modelling is more challenging in an urban environment where traffic can greatly influence travel times. We also expected to see a better correlation between MA and PA by respondents who do not have access to motorized transportation because we suppose access to motorized transports may induce important variability and complexity in mobility patterns, and increases the odds of choosing another health facility than the closest one. Those assumptions were confirmed by our correlation analyses: there is a stronger association between PA and MA for respondents living in households without access to motorized transportation, whatever the modelling method used.

Regarding the performance of each method, all countries taken together, our results show that Euclidean distance is more correlated to perceived accessibility than the other, more sophisticated methods. However, when stratified per country, results were very different, with Kernel density being the best correlated method for half of the countries. Such contrasting results suggest that none of the four methods tested outperform the others in the explanation of the perceived accessibility. In other words, more complex methods such as cost-distance do not show any benefit compared to simpler methods like Euclidean distance, when working with globally-consistent datasets. In particular, given that the cost-distance method that considers walking speed is strongly correlated to Euclidean distance (r_P_ = 0.994), using Euclidean distance is preferable, as the additional complexity in terms of input data, computing skills and resources for the cost-distance method is not compensated by a clear added value in terms of prediction accuracy. Cost-distance considering motorized speed did not show better results, even when only considering the motorized population.

We expected cost-distance methods to show better correlation results with perceived accessibility, given that these methods take geographic heterogeneities and obstacles into account to produce accessibility estimations. Those low correlation levels could be explained by multiple uncertainties arising from the friction map used to produce the motorized cost-distance maps. In these gridded speed maps, the spatial resolution is ~ 1 × 1 km and each cell receives a speed value from a road, if there is a road crossing the cell. This leads to some important travel-time overestimations, especially in urban areas. Moreover, road speed values are based on speed maximum limits, which can be an overestimation of the actual speed in many cases as traffic, road deterioration, the impassability of some roads during the rainy season, or the inability of a vehicle to reach that speed limit are not considered. All those elements can potentially explain why cost-distance maps considering motorized speed are not showing convincing correlation results at our scale of analysis. We can compare these conclusions with Baier et al. [4] who also identified better correlations between MA and PA in rural areas than in urban areas, in Germany. They also showed that a simple method, network distance to a physician, was as effective as the more complex FCA method. Boscoe et al. [5] and Al-Taiar et al. [3] also identified simple straight-line methods as efficient as cost-distance methods in USA and in Yemen, respectively. Our results confirm those tendencies in a sub-Saharan continental geographic context, with nuance brought to the inter-country variability of each method’s performance. Also note that we worked at medium spatial resolution, using globally-consistent friction maps at 1-km resolution. Results may be different at finer spatial resolution and using locally parameterized methods, using for example field measurements of speed values to produce the friction maps. Further analyses would be needed to estimate more precisely the impact of the spatial resolution on the results, especially as the impact is probably variable across space and according to the availability of finer input data.

The Kernel density method is showing puzzling results. We chose to add this method in order to evaluate the effect of having a method that is considering more than one health-center in the vicinity. However, this method has the best correlation values for most countries, when looking at per-country results, but the worst when looking at all data together. It looks like the stratification of results strongly influences the performance of this method, which could be due to the way the results of this method are distributed. The performance of the Kernel density method could be improved by optimizing the function used to generate the point-density surface and the maximum distance used for extrapolation, eventually using context-specific parameters. Kernel density correlation is also sensitive to the amount of population living outside of its distance threshold, which was set at 15 km here. For large countries with an important proportion of the population living at more than 15 km from a health facility, this creates many cluster points with null accessibility values, which may drag the correlation results down because of the absence of variability in the accessibility measure for this part of the population. This hypothesis could explain the low performance of the Kernel density method for Angola or Ethiopia, compared to the other methods. Based on our results, we suggest that this method is not the most appropriate for an evaluation of regional disparities in accessibility to health facilities, because of its unstable and difficult interpretation of results. However, the Kernel density method could be used in more specific accessibility evaluations when the focus is to evaluate the amount of health facilities surrounding a population.

To measure the perceived accessibility, we used a question in the DHS survey for women, which is the following: “When you are sick and that you need medical advice or treatment is the distance an obstacle to you, yes or no.” This question could be considered as unclear and may be subject to many biases coming from socio-cultural and cognitive factors, which could influence the way respondents answer it. Distance being an obstacle or not is a vague statement and is subject to individual interpretation [6]. The translation of essential words such as “distance” or “obstacle” in the respondents native language could also influence the answers [22]. We believe however that the respondent dataset is large enough (n = 206 259) to evaluate perceived spatial accessibility patterns. Another cultural bias that could lead to interpretation errors of our results is the gender effect, given that the perceived accessibility is only available for women respondents. Access to motorized transportation and mobility needs can be different between men and women in SSA [1, 24], which might bias the perceived accessibility of our respondent group. Further studies should evaluate the relationship between accessibility prediction performance and gender.

We evaluated the error induced by the DHS random displacement by reproducing that displacement 30 times on the cluster points datasets and see how it affects correlation results. We saw in the results that the impact on correlation is not negligible, but is not important enough to hide the effect of the country or the effect of the methods on correlation results. Our results therefore suggest that the random displacement applied to cluster survey points for privacy reasons do not prevent large-scale accessibility analyses as done here. Furthermore, we have to keep in mind that the relationship between PA and MA may be underestimated due to such displacements.

The correlation results are likely to be strongly influenced by the exhaustiveness of the health facility dataset used as input. Here we used the data assembled in by Maina et al. [19], which was produced through a rigorous data collection protocol with a combination of public and private sources. However, the quality is variable from a country to another, and we suspect that missing health facilities may lead to an important offset between MA and PA.

## Conclusions

The goal of this paper was to evaluate how four commonly used accessibility models relate to perceived accessibility, in 12 different countries of sub-Saharan Africa. Our key finding is that we found no evidence that cost-distance or Kernel density methods were better at explaining the population perceived accessibility than the straightforward Euclidean distance method, when using global input datasets with ~ 1 × 1 km spatial resolution. We suggest that cost-distance methods might become beneficial when used with accurate field-based input data to construct friction maps, such as transportation speeds on different terrains or exhaustive health provider locations. We also highlight that accessibility models are better at explaining perceived accessibility in rural areas and by populations who do not have access to motorized transportation. Globally, we also show that accessibility models have a low correlation level with perceived accessibility, which highlights the importance of including spatially-finer or individual data to predict the experienced accessibility of a population. Further studies are needed to evaluate how locally parameterized methods, such as cost-distance based on field measurements of speed values and with fine spatial resolution friction maps, would benefit perceived accessibility predictions compared to global methods.

## Data Availability

All data used in this study are freely available to the public and are referenced in the paper.

## Abbreviations

CD-M: Cost-distance motorized
CD-W: Cost-distance walking
DHS: Demographic and health surveys
ED: Euclidian distance
GIS: Geographical information system
GPS: Global positioning system
KD: Kernel density
MA: Modelled accessibility
PA: Perceived accessibility
r_P_: Pearson correlation coefficient
r_S_: Spearman correlation coefficient
SSA: Sub-Saharan Africa
